# New Discovery of Myeloid-Derived Suppressor Cell’s Tale on Viral Infection and COVID-19

**DOI:** 10.3389/fimmu.2022.842535

**Published:** 2022-02-03

**Authors:** Soo-Jeung Park, Da-eun Nam, Hae Chang Seong, Young S. Hahn

**Affiliations:** ^1^Beirne B. Carter Center for Immunology Research, University of Virginia, Charlottesville, VA, United States; ^2^Department of Microbiology, Immunology and Cancer Biology, University of Virginia, Charlottesville, VA, United States

**Keywords:** MDSC, COVID-19, immune regulation, biomarkers, therapeutics

## Abstract

Myeloid-derived suppressor cells (MDSCs) are generated under biological stress such as cancer, inflammatory tissue damage, and viral infection. In recent years, with occurrence of global infectious diseases, new discovery on MDSCs functions has been significantly expanded during viral infection and COVID-19. For a successful viral infection, pathogens viruses develop immune evasion strategies to avoid immune recognition. Numerous viruses induce the differentiation and expansion of MDSCs in order to suppress host immune responses including natural killer cells, antigen presenting cells, and T-cells. Moreover, MDSCs play an important role in regulation of immunopathogenesis by balancing viral infection and tissue damage. In this review article, we describe the overview of immunomodulation and genetic regulation of MDSCs during viral infection in the animal model and human studies. In addition, we include up-to-date review of role of MDSCs in SARS-CoV-2 infection and COVID-19. Finally, we discuss potential therapeutics targeting MDSCs.

## Introduction

Coronavirus Induced Disease 2019 (COVID-19) caused by Severe Acute Respiratory Syndrome Corona Virus (SARS-CoV)-2 started in the Hubei province, China in 2019 (1). In the past two years, despite the efforts of many countries in response to the global pandemic situation, more than 260 million COVID-19 cases have been confirmed and over 5.2 million deaths have been reported to the World Health Organization (2). Patients infected with SARS-CoV-2 show various pathologies ranging from asymptomatic to mild, moderate, severe, and fulminant symtoms. Among them, critical pathology is followed by complications such as respiratory failure, myocarditis, sepsis, and various organ failures (3, 4).

It has been reported that the onset of COVID is not only directly affected by the virus, but also affected by immune responses such as cytokine storm syndrome, neutropenia, and lymphopenia (5, 6). However, the main potential targets for therapeutic purposes in relation to these immune mechanisms still remain challenging. Reduced function and depletion of CD8^+^ T cells were observed in patients with severe symptoms of COVID-19 (7, 8), CD4^+^ T cells have been reported to be important for patient recovery and protective immunity against SARS-CoV-2 (9, 10).

So far, immunological studies for COVID-19 individuals have mainly focused on innate and adaptive immune responses. Several studies report how the induction of MDSC and its role affect the progression of COVID-19 (11–14). Accordingly, studies confirm the role of the MDSC subset in severity of COVID-19 progression and provide potential therapeutic targets for COVID-19 (15–17). Further research identifying new biomarkers could be critical in developing therapeutics for disease prevention and amelioration. In this review, we discuss the latest studies focusing on the immunoregulating properties of MDSC and with biomarkers that may influence the intervention in treatment of COVID-19.

## Dysregulated Immune Response in COVID-19

Since the outbreak of COVID-19, numerous publications related to immune responses and immunopathogenesis have been reported. The pathophysiology of COVID-19 has been listed as a complex state according to age, sex, pregnancy, presence of underlying disease, etc. (18–20). In the innate immune system, similar to Middle East respiratory syndrome (MERS)-CoV, SARS-CoV-2 modifies the signaling of TRAF3 and RNA sensor adapter molecules (MAVS) through proteins such as PLpro and inhibits type I interferon (IFN-1) production (21, 22). Antagonism of IFN-1 production aids viral replication, promotes release of pyroptotic products, and induces additional inflammatory responses (23). Pyroptosis is a form of programmed cell death within inflammatory cells (24) and is mediated by the production of IL-1β during SARS-CoV-2 infection (25).

Severe COVID-19 patients showed impaired IFN-1 signaling compared to mild patients, and developed an inappropriate inflammatory state due to early delay in IFN-1 expression and activation of pro-inflammatory cytokines (IL-1, IL-6, IL-8, MCP-1, and CXCL-10) (26–28). In addition, there were high viral titers and accumulation of monocyte-derived macrophages and neutrophils in the lungs. This condition leads to a systemic inflammatory response and cytokine storm syndrome *via* a massive release of cytokines. In addition, the risk of COVID-19 may increase or decrease differently depending on the number and activity of natural killer (NK) cells. Healthy children have been reported to have more NK cells than adults and the elderly (29), which might explain why children are expected to have a better defense against SARS-CoV-2. According to a recent study, the number of NK cells in adults with severe COVID-19 was reported to be low, and the activated form of CD56^low^ NK cells increased to generate cytokines (30, 31).

In terms of the adaptive immune system, inefficient innate immune responses in SARS-CoV-2 infection lead to dampen adaptive immune responses and exacerbate inflammation (32). Pro-inflammatory cytokines induce the expansion of CD4^+^ and CD8^+^ T cells, decrease regulatory T, and lead to activation of Th1-type and B cells. When patients with underlying severe disease are infected with SARS-CoV-2, the number of lymphocytes is decreased while the blood levels of CD4, CD8, and regulatory T cells were also significantly lower than those in patients with mild disease. At this time, monocytes, macrophages, and T cells are accumulated in the lungs, and T cells migrate from the blood to these tissues to regulate the depletion of blood lymphocytes (23). In addition, a recent study showed that the lymphocyte counts of children infected with SARS-CoV-2 remained at a steady normal level compared to adults, and thus had less negative effect on immunomodulation (33).

Extensive studies on analyzing immune responses in COVID-19 patients show that the number of T or B lymphocytes, DC, NK cells, and HLA-DR^high^ cells are reduced in patients with severe symptoms (34–36). Additionally, severe COVID-19-associated hyperinflammatory syndromes have been reported that they originate from a host innate immune response (37). Studies of transcriptome, proteomic, and epigenomics have revealed a wide range of functional impairments, including marked neutrophil hyperactivation symptoms in severe COVID-19 (38–41). Collectively, COVID-19 caused by SARS-CoV-2 is associated with a failure of innate and adaptive immune system regulation due to changes in other immune cells associated with a decrease in adaptive T cells. Although the link between the immune systems is still only partially explained, studies on MDSC, have been risen significantly and may provide explanation for dysregulated immune responses in COVID-19.

## MDSC’s Immunoregulatory Function in Viral Infection

### MDSC Phenotypes

Myeloid-derived suppressor cells (MDSCs) are defined as a heterogeneous population of immature bone marrow cells that suppress T cell responses, and was first described in a mouse model of lung cancer in 1987. Together with myeloid progenitor cells, they have the ability to suppress the immune responses at the forefront of viral infection (42). It has been reported that these cells have changed research fields related to cancer, inflammation, and immune response over the past 30 years, and even serve as a marker for distinguishing disease progression (43–45). MDSCs are mainly classified into two distinct group–neutrophilic/granulocytic (PMN)-MDSCs and monocytic (M)-MDSCs (46, 47) (**Table 1**). Granulocytic MDSCs have multi-lobed nuclei similar to polymorphonuclear cells, and monocytic MDSCs have a single, round nucleus; therefore, they look similar to monocytes (48). The morphological heterogeneity of these cells depends on the expression of Gr1, and the Gr1-specific antibody binds to both Ly6G and Ly6C, which are myeloid lineage differentiation antigens. Granulocyte and monocyte MDSCs of mice have phenotypes of CD11b^+^Ly6G^+^Ly6C^low^ and CD11b^+^Ly6G^-^Ly6C^high^, respectively (49). Human MDSCs are mainly identified as phenotypic markers Lin^−^HLA-DR^−^CD33^+^ or CD11b^+^CD14^−^CD33^+^ (46). In early studies on MDSC, the target of MDSC-mediated suppression was mainly T cells. After that, research on MDSC was gradually expanded, and it was additionally reported that it modulates innate immune cells such as NK cells, dendritic cells (DCs), and macrophages as well as adaptive immune cells such as B cells (50–52).

**Table 1:** Two categories of myeloid-derived suppressor cells and functions.

Type of MDSC	Markers		Immunosuppression mediators and mechanisms
	Murine	Human	
PMN-MDSCs	CD11b^+^Ly6G^+^Ly6C^low^	CD11b^+^CD14^-^CD15^+^HLADR^-^	Suppressive immune responses, ROS, ARG1, CD33, and CD66b
	CD11b^+^GR-1^high^	CD11b^+^CD14^-^CD66b^+^	
		LOX-1^+^	
M-MDSC	CD11b^+^Ly6G^-^Ly6C^high^	CD11b^+^CD14^+^CD15^-^HLADR^low/-^	Suppressive T cell responses, NO, iNOS, ARG1, pSTAT3, S100A8/9, IL-4R, TGF-1β, HLA-DR, and IRF8
	CD11b^+^GR-1^low^		

### Immunosuppressive Function of MDSCs

Soluble factors related to MDSC function include reactive oxygen species (ROS), inducible nitric oxide synthase (iNOS), and arginase-1. Each of these key mediators independently attenuates the host immune response. Granulocytic MDSCs mainly use ROS generated by NADPH oxidase to cause immunosuppression. Monocytic MDSCs use iNOS to generate nitric oxide (NO) (48, 53, 54). iNOS nitrosylates T-cell receptor (TCR) together with arginase-1 to generate reactive nitrogen-oxide species that inhibit T-cell or induce apoptosis (55). Interestingly, both granulocytic and monocytic MDSCs utilize the action of arginase-1 to deplete L-arginine, which is required for T cell proliferation and function (56, 57). Effects of T-helper (Th)1 and Th2 cytokines, such as interleukin (IL)-2, IL-4, IL-13, and interferon (IFN)-γ on aginase-1 led to the identification of crosstalk between MDSCs and T cells (58–61).

MDSCs from hepatocellular carcinoma patients have been shown to inhibit NK cell cytotoxicity and IFN-γ release (62). This was an arginase-1 independent, contact-dependent suppression effect that required the expression of NKp30, a receptor for NK cells. In the case of MDSCs expanded from tumor mice, membrane-bound transforming growth factor-β1 (TGF-β1) inhibited NK cell cytotoxicity, IFN-γ production, and the expression of the activating receptor NKG2D (63). In addition, MDSCs cause differentiation of immature DCs in cancer and limit the immune response by inhibiting the antigen uptake ability of DCs (64–66).

During viral infection, similar increases in PMN- and M-MDSCs are initially observed in acute and chronic infection models, but rapidly return to baseline levels in acute cases. In chronic infection, it has been reported that it takes quite a long time to return to the baseline level. In the case of the mouse acute infection model, the inhibitory activity of M-MDSC did not appear at any time point. In the case of chronic infection model, it became more prominent from the 7th day to the 14th day after infection and decreased on the 30th day, but it was still detected. Recently, it has been reported that the ER stress response is essential for the inhibitory activity of M-MDSC in viral infection, and it is known that the acquisition of the most potent inhibitory activity is mediated by IFN-γ signaling (67).

### Factors Involved on MDSCs Generation

Selective mediators have been shown to be responsible for the generation of MDSCs. Prostaglandin E2 (PGE2) exerts numerous biological actions, including anti-inflammatory and pro-inflammatory and is a key mediator for MDSC generation. Administration of PGE2 blocks DC differentiation and allows myeloid progenitor cells to acquire the characteristics of MDSC (68). In addition, anti-inflammatory mediators such as NOS2, iNOS, indolamine-2,3-deoxygenase (IDO), and IL-10 are secreted between PGE2 and cyclooxygenase 2 (COX-2), and their function as MDSCs regulating immunosuppression has been reported (69, 70). MDSCs suppress T cell effector function *via* co-expression of arginase1 and NOS1. When they are added to the co-culture of MDSC and activated T cells, T cell function is completely restored (71). Since the expression level of PGE2 is elevated when tumors form, the inhibitory effect of COX-2 is considered to partially affect the reduction in MDSC production. Among the many factors that can induce MDSC production, IL-1β further stimulates the recruitment of MDSCs in the non-tumor state (72). S100A9 increases the immunosuppressive ability of MDSCs by increasing the expression of nuclear factor-κB (NF-κB) dependent arginase by binding to the receptor for advanced glycation end products (RAGE) (46, 73).

**Figure 1:**
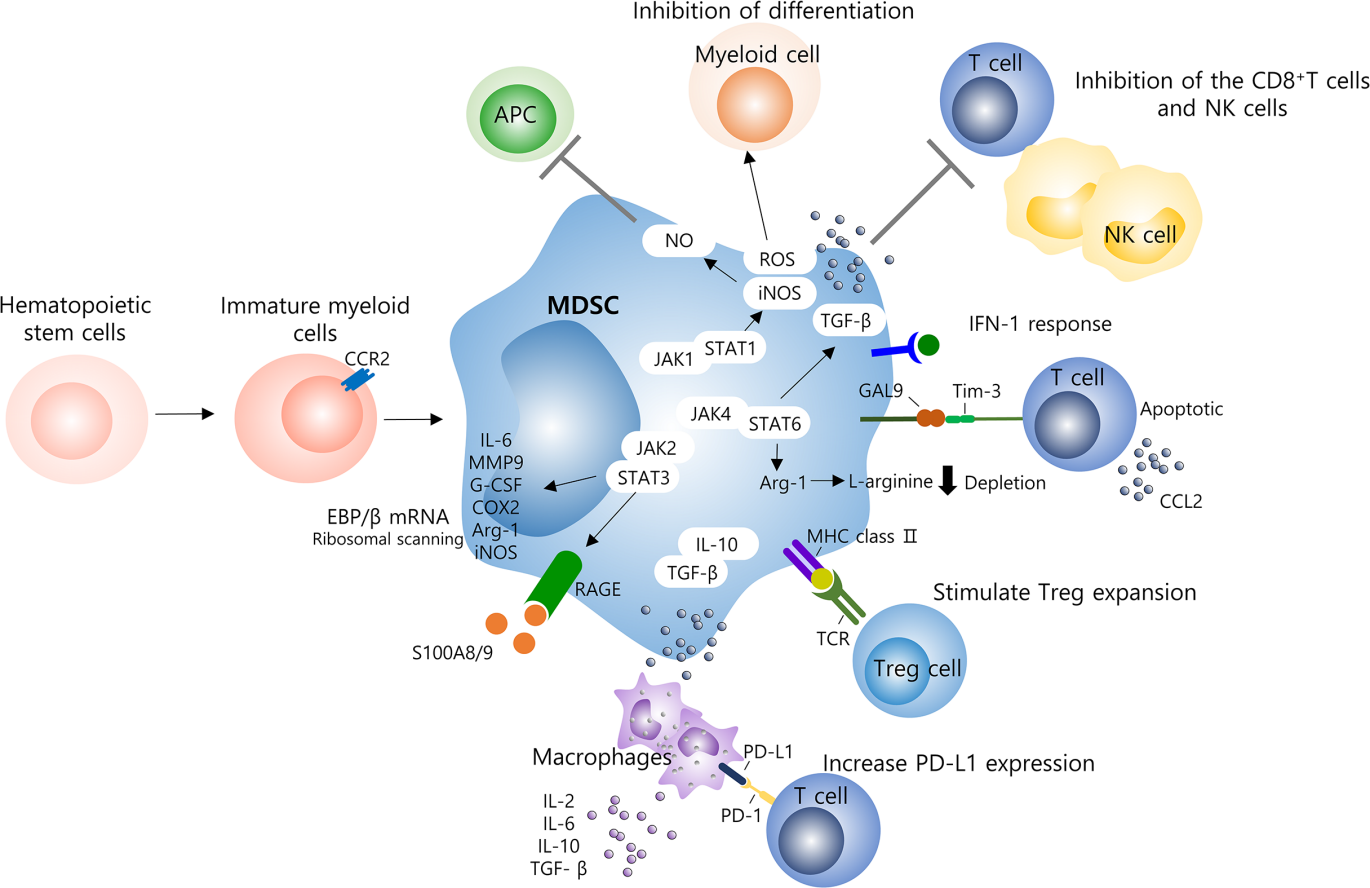
Molecular mechanisms of MDSCs on the immune system. Several mechanisms contribute to MDSC-induced immune suppression and hyperinflammatory activation in viral infection and including those with COVID-19 patients. Specially, MDSCs are able to suppress T cells, NK cells, and other myeloid cells function. T cells are mainly inhibited through the production of ROS or depletion of L-arginine, and the delayed production of IFN-1 seems to result in the continuous accumulation of MDSCs into the lungs. Several signaling pathways, such as STAT1/3/6, are involved, to increase levels of immunosuppressive factors such as ROS, iNOS, NO, and Arg-1, which inhibit T cell responses. High levels of PD-L1 found in MDSCs and macrophages can reduce the activation of antigen-specific T cells by binding to the PD-1 receptor on T cells. In addition, the release of IL-10 and TGF-β by MDSC induces additional inflammatory system of macrophages by recruiting lymphocytes and granulocytes as well as inflammatory monocytes. MDSCs, myeloid derived suppressor cells; NK, natural killer cell; ROS, reactive oxygen species; TGF-β, transforming growth factor-β; APC, antigen presenting cells; NO, nitric oxide; STAT, signal transducer and activator of transcription; JAK, janus activated kinase; IL, interleukin; MMP, matrix metalloporteinases; G-CSF, granulocyte colony stimulating factor; COX-2, cyclooxygenase 2; Arg, arginine.

To date, MDSCs have also been reported in numerous non-cancer pathologies, including viral, parasitic, bacterial, and fungal infections (74, 75). The functional role of accumulated MDSCs in most infectious diseases is to inhibit host defense and regulate inflammatory cytokines such as TNF-α, IL-1β, and IL-6 (76). Some studies have also mentioned a detrimental role of MDSC, but the study model, pathogen, disease stage, and T cell ratio show different results (77–79). In relation to the study of MDSC viral infection, induction of MDSC expansion of viruses such as hepatitis B or C virus, Epstein-Barr virus, human papillomavirus, influenza, and SARS-CoV-2 has been reported, and these induce virus persistence; nonetheless, evidence of MDSC’s tissue damage protective role has also been reported in other studies (80–82). In these various inhibitory mechanisms, the activity of MDSCs effectively interferes with enhancing tumor and non-tumor immune responses (**Figure 1**).

The factors involved in the production and suppression of MDSC in various disease states include stem cell factor (SCF), HIF-1α, IL-6, macrophage colony stimulating factor (M-CSF), signal transducer and activator of transcription 3 (STAT3), myeloid-related protein S100A9, and IL-1β (51, 52, 70, 83, 84). In these microenvironmental factors, MDSCs go through a journey to the site of immune response to exert an immunosuppressive effect. In particular, the efflux from the blood to the tumor depends on CXCR4 and also affects the chemotaxis of mature myeloid cells (85). Another function of MDSC is epigenetic regulation. Histone deacetylase-2 (HDAC-2), which has been studied for a long time as a cancer treatment agent, converted monocyte MDSC to granulocyte MDSC, and the mechanism was suggested. In addition, HDAC-11 suppressed the expression of IL-10, an immunosuppressive cytokine, in chromosomal modification due to the action of tumor-derived factors (TDF) infiltrating MDSC (86). Meanwhile, the DNA methylation inhibitor zebularine decreased the expression of IDO, a potent immunosuppressive mediator of MDSC (87). This may support the epigenetic function of MDSCs regardless of acetylation or methylation.

### MDSCs Function and Biomarkers in COVID-19

MDSCs are innate immune cells that can be increased in activity by infection-causing factors as previously described for various viral, parasitic, and bacterial infections, and also regulate the adaptive immune system. Several studies related to COVID-19 have reported that the high frequency of MDSC is associated with symptoms of severe disease and appears in the form of myeloid cell compartments that are difficult to control. Expansion of MDSC that occur in blood of severe COVID-19 patients had a close effect on lymphopenia and enhanced arginase activity (88). In particular, the ratio of MDSC to effector CD8^+^ T cells was increased in patients with severe pneumonia accompanied by acute respiratory distress syndrome (ARDS). MDSC frequencies in total circulating mononuclear cells ranging from mild to severe cases were recorded at a maximum of 25% and 90%, respectively (89). In addition, MDSC is involved in not only the inhibitory effect on T cell proliferation and activation, but also functional impairment of NK cells, B cell inhibition, Treg expansion induction, and downregulation of cytokine production by macrophages (89, 90). Markers suggesting granulocytes, such as eosinophils, neutrophils, and basophils, were highly expressed in COVID-19 patients, predicting the activity of granulocytic MDSCs. Decrease in the expression of granulocytes identified by the integrin CD11b, increase in the number of neutrophils identified in CD15^+^CD16^+^, and down regulation of Th2-related CRTH2 in eosinophils and basophils were established as the signature of COVID-19. In addition, the appearance of PD-L1 checkpoint expression in eosinophils and basophils was found to be related to severity (91). In the immune cell metabolism program of COVID-19 patients, voltage-dependent anion channel 1 (VDAC1) was expressed in the T cell population, which is associated with mitochondrial dysfunction and apoptosis. This may provide a means to predict disease severity, follow-up, and design metabolic therapy regimens (92).

#### PMN-MDSCs in COVID-19

PMN-MDSC was expanded during COVID-19, especially in patients requiring intensive care. A positive correlation was found between PMN-MDSC and the concentration level of inflammatory cytokines (IL-1β, IL-6, IL-8, and TNF-α) in the blood (93, 94). Inflammatory cytokines play a central role in inducing the expansion of MDSCs (46). The expression level of lectin-type oxidized LDL receptor 1 (LOX-1) was suggested as a marker to distinguish a subset of MDSCs with strong immunosuppressive ability in patients suffering from ARDS (16). In addition, a significant increase in hexokinase II^+^ PMN-MDSC was confirmed in severe COVID-19 patients with moderate or severe disease. In mild COVID-19 patients, IFN-stimulating inflammatory HLA-DR^hi^CD11c^hi^ monocytes increased, and IFN-1 deficiency was confirmed in severe patients. On the other hand, the high frequency of monocytes in HLA-DR^low^ and neutrophils in CD10^low^CD101CXCR4^+/-^ suggest emergent myelopoiesis as immunosuppressive markers in the blood and lungs of severe patients (95–101). Reductions in MDSCs during the COVID-19 recovery phase were associated with increases in inflammatory cytokines in the patient’s blood, including decreases in TGF-β (88). A multivariate regression analysis showed an association between the PMN-MDSC rate and fatal disease state, and the frequency of PMN-MDSC was higher in the non-survivors group than that in the recovered group (93). A recent study reported a significant correlation between C-reactive protein, ferritin, and lactate dehydrogenase levels and MDSC in patients with COVID-19. This indicates that immature PMN-MDSCs are associated with disease severity (102). Expansion of PMN-MDSCs and immature neutrophils in severe COVID-19 conditions indicates Th1 cell suppression and an increase in the frequency of Th17 cells due to strong polar migration to Th17 cells (103).

#### M-MDSC in COVID-19

M-MDSCs that have been mainly identified in PBMCs from acute COVID-19 patients, are associated with disease severity, and also suppress T cell responses (17). They are characterized by expressing VDAC and carnitine palmitoyltransferase I. M-MDSC isolated from COVID-19 patient inhibited T cell proliferation and IFN-γ production through an Arg-1-dependent mechanism, and increased Arg-1 and IL-6 levels (104). In an *in vitro* study that tested T cell proliferation, arginine supplementation helped restore T cell proliferation in patients with COVID-19, which had been reduced (88). In addition, the monocyte distribution width (MDW), which has recently emerged as a promising early biomarker of sepsis, has been considered as a key mediator of hyperinflammatory disorders in severe COVID-19 conditions. High MDW values have been reported to be associated with prognostic lethal outcome in COVID-19 patients (105).

The presence of neutrophils and macrophages was confirmed in the bronchoalveolar lavage fluid (BALF) from patients with severe COVID-19, while large amounts of cytokines and chemokines were secreted. Among the gene signatures identified in single-cell RNA sequencing (scRNAseq) data, gene sinatures such as H3F3B, IFITM1/2, SAT1, and S100A8 are associated with neutrophils, and CCL2/3/8, KLF6, and SPP1 are associated with macrophages (106, 107). This was the similar result as the high level of S100A8/9 found in the plasma of severe patients (98). High levels of proinflammatory cytokines and chemokines such as CXCL8, IL-6, and IL-10 were associated with upregulation of the monocyte compartment (108, 109). Another additional serum chemokines and cytokines (IL-6, IFN-λ3, IP-10, CXCL9, CXCR1/2/4 and CCL17), virus-sensing TLRs, HIF1α, and several genes involved in various metabolic regulation were identified in COVID-19 (110, 111). Furthermore, it was reported that soluble triggering receptor expressed on myeloid cells and an IL-6-based algorithm could serve as a very sensitive marker for early discrimination among patients with adverse reactions among COVID-19 patients (112). Although more research is needed, one of the markers that can predict the correct mortality rate among COVID-19 ICU patients is mid-regional pro-adrenomedullin (MR-proADM): it presented as high levels in non-survivors (113). In addition, several recent studies have shown new markers such as neutrophil-to-lymphocyte ratio, neutrophil-to-platelet ratio, uric acid level, total antioxidant capacity, eosinophil/PMN ratio, high-density lipoprotein, and apoprotein A1 (114–119).

In summary, monocytes and segmented neutrophils from peripheral blood migrate to immature myeloid cell candidates due to the elevation of cytokines and pro-inflammatory mediators during COVID-19, demonstrating the generation of emergency myeloid cells. Bone marrow cells identified in severe COVID-19 conditions are a subset of the primary immune cells that have initiated their activity, and studies on their ratio, inflammation, and identification of chemotactic genes will lead to potential diagnostics and therapeutics. Many studies related to COVID-19 have addressed the roles of MDSCs and their subsets, suggesting their selection as biomarkers for immune dysregulation in COVID-19 (120). This is clearly clinically meaningful given its correlation with disease. Candidates for newly added biomarkers related to COVID-19 are shown in **Table 2**.

**Table 2:** Candidates for biomarkers to identify COVID-19 severity.

Biomarkers	Responses of each markers in COVID-19	References
CD15+CD16+CD11b^low^	Increased	(91)
PD-L1	Increased	(91)
VDAC1	Increased	(92)
LOX-1	Increased	(16)
Hexokinase II+	Increased	(17)
T cell and NK cell ratio	Increased	(15, 88)
HLA-DR^hi^CD11c^hi^	Increased	(95)
IFN-1	Decreased	(96)
HLA-DR^low^	Increased	(98)
Calprotectin (S100A8/9)	Increased	(98)
CD10^low^CD101CXCR4^+/-^	Increased	(95, 96)
TGF-β	Increased	(88)
C-reactive protein, ferritin, and lactate dehydrogenase level	Increased	(102)
Arg-1 and IL-6 level	Increased	(104)
VDAC and carnitine palmitoyltransferase I	Increased	(17)
MDW	Increased	(105)
CXCL8 and IL-10 level	Increased	(108, 109)
IFN-λ3, IP-10, CXCL9, and CCL17 level	Increased	(110, 121)
HIF1α	Increased	(111)
MR-proADM	Increased	(113)
LDH, D-dimer	Increased	(122, 123)
Neutrophil-to-lymphocyte ratio	Increased	(124)
Neutrophil-to-platelet ratio	Increased	(114)
Uric acid level	Increased	(115)
Total antioxidant capacity	Decreased	(116)
Eosinophil/PMN ratio	Decreased	(117)
High-density lipoprotein	Decreased	(118)
Apoprotein A1	Decreased	(119)

## Potential Therapeutics Targeting MDSC in COVID-19

Based on the expansion of MDSC in COVID-19, several molecular mechanisms for MDSC differentiation have been elucidated, making it possible to target and develop therapeutic agents. As cancer therapy, MDSC removal is beneficial to boost anti-tumor immunity such that chemotherapy reduces MDSC-mediated inhibition of T cells (125, 126). Rather this therapy was able to concert MDSCs into pro-inflammatory cells and disrupt tumor growth (127). Given the immunophenotype and suppression mechanism of the existing tumor microenvironment (TME) are diverse, it is challenging to target various human MDSC types (128). By using information gained from cancer studies, MDSCs-target therapeutic strategies can be applied to COVID-19 (70, 129). **Table 3** summarizes the core of MDSCs targeting strategies for disease control and inhibition of MDSCs activity identified so far and the candidates applicable to COVID-19. Various types of drugs have been reported as 1) drugs that differentiate MDSCs into mature myeloid cells, 2) drugs that interfere with MDSC maturation from cell precursors, 3) drugs that reduce MDSC accumulation in peripheral organs, and 4) drugs that affect MDSC inhibitory function (120).

**Table 3:** Potential therapeutic candidates for targeting MDSCs.

Strategy	Agents	References
Promote MDSCs differentiation to increase mature leukocytes and tumor-specific T cells	ATRA, 1α,25-dihydroxyvitamin D3, DNA-methylating agent 5-azacytidine, CpG oligonucleotides, chemotherapeutic agents (paclitaxel and docetaxel), RUNX1, casein kinase inhibitor (tetrabromocinnamic acid)	(130–136)
Directly block MDSC supprression of T cells	COX-2 inhibitors, Phosphodiesterase type 5 inhibitors (tadalafil and sildenafil)	(137–142)
Inhibit migration of myeloid cells from the bone marrow to the tumor microenvironment or peripheral lymphoid organs	CXCR2, CXCR4, CSF1R, and CCR2/5 inhibitors	(143–146)
Inhibit the production of MDSCs from progenitors or induce apoptosis of circulating MDSCs	5-fluorouracil, gemcitabine, sunitinib, and zolendronate	(125, 126, 147–149)
Block the production of TDF and its reach into the bone marrow	Targeting the IL-6 receptor (tocilizumab) and HDAC-11	(86, 150)
Cytokines targeting MDSC	S100A8/A9 inhibitor (paquinimod)	(151, 152)

First, as a potential MDSC target, COX-2 inhibitors are useful because of significant role of the PGE-COX-2 axis in MDSC generation. COX-2 inhibitors inhibit the migration of MDSCs to tumor sites and reduce the incidence of several cancers by regulating transcriptomes (137–139). Prostaglandin D2 (PGD2) plays a role as a key mediator for lymphopenia in a recent COVID-19 study, and induces upregulation of M-MDSC through the DP2 receptor in group 2 innate lymphoid cells. Targeting PGD2/DP2 signaling using a related receptor antagonist (ramatroban) has been consedered as a therapy to address immune dysfunction and lymphopenia in COVID-19 (153).

Second, a phosphodiesterase-5 inhibitor (tadalafil) approved by the Federal Drug Administration (FDA) can inhibit MDSC by inducing downregulation of Arg1 and iNOS activity in several preclinical models (140–142). In a study using an animal model, tadalafil decreased the levels of glutamic oxaloacetic transaminase, an enzyme that promotes carbohydrate and protein metabolism in aflatoxin-induced liver cancer cells (154). Similarly, all-trans retinoic acid (ATRA), which has been previously used as a treatment for acute promyelocytic leukemia, induces MDSD differentiation, allowing NKT cells to mature into a state where they can be helped. ATRA also induces the expression of glutathione synthase, which leads to the production of glutathione, a ROS neutralizing agent (130). 1α,25-dihydroxyvitamin D3 (calcitriol) is able to inhibit IL-6-stimulated MDSC proliferation in a mouse esophageal squamous cell carcinoma model (131). This treatment can be extended to treatment of other diseases. Strong pathogen molecular patterns, such as CpG oligonucleotides and paclitaxel, have also been shown to differentiate MDSCs into mature myeloid cells (132, 133).

Third, potential drug can be designed toward aiming at the migration/recruitment of myeloid cells among treatment strategies in order to recover COVID-19-related hyperinflammation and the resulting immunomodulatory disorders (155). CXCR2 and CCR2/5 inhibitors are known to decrease the migration of MDSCs from the bone marrow to the circulation (143, 144). The importance of inhibiting MDSC proliferation and migration to TME with strategies such as anti-CXCR2 monoclonal antibody has also been reported (145). Leronlimab (CCR5 blocking antibody) decreased plasma IL-6 levels, restored the CD4/CD8 ratio, and induced a decrease in SARS-CoV-2 plasma viremia (156). The previously mentioned alamin S100A8 is strongly induced in COVID-19 patients and SARS-CoV-2 infected models. Paquinimod, known as an inhibitor of S100A8/9, induced a decrease in viral load in mice infected with SARS-CoV-2, resulting in relief from pneumonia (151). Although the number of neutrophils increases in the onset of COVID-19 and is accompanied by uncontrollable pathological damage, paquinimod decreased the number of neutrophils. The treatment of this drug can be suggested as a method for therapeutic purposes while simultaneously detecting abnormal changes in S100A8/9 and neutrophils in the COVID-19 state (151).

Lastly, drug can be designed to regulate metabolism of MDSCs. The inhibition of arginase-1 or supplementation of arginine in severe COVID-19 patients could be a treatment to restore depleted arginine and impaired T cell function (157). Moreover, reprogramming of MDSCs targeting epigenetics based on immune metabolism is also expected to solve the pulmonary inflammatory state of COVID-19 (158). This diverse list of drugs capable of manipulating MDSC populations could play an important role in MDSC-related treatment modalities in chronic inflammation, cancer, as well as in COVID-19.

## Conclusions

MDSCs plays a pivotal role in regulating the innate immunity and adaptive immunity. Dysregulated immune response and MDSCs expansion have been reported in COVID-19 and other viral infections. The removal of MDSC leads to an increase in the immune response against the viral infection. Thereby, active research has been conducted to identify various MDSC phenotypic markers and discover therapeutic agents targeting MDSCs. Despite of the detrimental role of MDSCs in human inflammatory diseases, MDSCs are resistant to allograft transplantation and autoimmune diseases, limits the inflammatory damaging, and shows a tendency to return to a non-inflammatory state by homeostasis. There are still few research reports that MDSC directly inhibits hyperinflammation and helps clinical recovery, but additional studies are needed because the possibility cannot be ruled out. In this regard, it is necessary to keep the tolerogenic properties of MDSC in mind to develop MDSC-targeted therapeutics (81).

In patients infected with COVID-19, both PMN-MDSC and M-MDSC accumulation and their expansion have been confirmed. Thereby, various markers for identifying MDSCs are important in the development of diagnostic systems. It can also open up several possibilities for treatment by targeting immunosuppressive function of MDSCs. The correlation between BALF and serum MDSC frequency and clinical biomarkers will facilitate the consideration and selection of future therapeutics. In many of these studies, the severity of COVID-19 was clinically evaluated using metabolites, cytokines, chemokines, and several proteins related to various mechanisms such as inflammation and apoptosis. Candidates considered as therapeutic agents for COVID-19 were typically presented as specific cytokine inhibitors or immunomodulatory agents. Although official approval of future treatments will be necessary, reports on various therapeutic approaches and treatment prognosis for approved drugs will still be required.

## Author Contributions

S-JP contributed to manuscript research and writing. D-eN contributed to manuscript research and writing. HS contributed to manuscript writing and review. YH contribute to manuscript supervision, writing, and review. All authors contributed to the article and approved the submitted version.

## Funding

This work was supported by NIH Grant (DK122737 to YH).

## Conflict of Interest

The authors declare that the research was conducted in the absence of any commercial or financial relationships that could be construed as a potential conflict of interest.

## Publisher’s Note

All claims expressed in this article are solely those of the authors and do not necessarily represent those of their affiliated organizations, or those of the publisher, the editors and the reviewers. Any product that may be evaluated in this article, or claim that may be made by its manufacturer, is not guaranteed or endorsed by the publisher.
